# CHADS_**2**_ Scores in the Prediction of Major Adverse Cardiovascular Events in Patients with Cushing's Syndrome

**DOI:** 10.1155/2014/138653

**Published:** 2014-07-01

**Authors:** Yuh-Feng Wang, Mei-Hua Chuang, Tzyy-Ling Chuang, Kung-Yung Huang, Shaw-Ruey Lyu, Chih-Yuan Huang, Ching-Chih Lee

**Affiliations:** ^1^Department of Nuclear Medicine, Dalin Tzu Chi General Hospital, Buddhist Tzu Chi Medical Foundation, Chiayi, Taiwan; ^2^School of Medicine, Tzu Chi University, Hualian, Taiwan; ^3^Department of Pharmacy, Dalin Tzu Chi Hospital, Buddhist Tzu Chi Medical Foundation, Chiayi, Taiwan; ^4^Division of Rheumatology, Department of Internal Medicine, Dalin Tzu Chi Hospital, Buddhist Tzu Chi Medical Foundation, Chiayi, Taiwan; ^5^Department of Education, Dalin Tzu Chi General Hospital, No. 2 Ming-Sheng Road, Dalin Town, Chiayi 62247, Taiwan; ^6^Division of Nephrology, Department of Internal Medicine, Dalin Tzu Chi Hospital, Buddhist Tzu Chi Medical Foundation, No. 2 Ming-Sheng Road, Dalin Town, Chiayi 62247, Taiwan; ^7^Cancer Center, Dalin Tzu Chi Hospital, Buddhist Tzu Chi Medical Foundation, Chiayi, Taiwan; ^8^Department of Otolaryngology, Dalin Tzu Chi Hospital, Buddhist Tzu Chi Medical Foundation, Chiayi, Taiwan; ^9^Center for Clinical Epidemiology and Biostatistics, Dalin Tzu Chi General Hospital, No. 2 Ming-Sheng Road, Dalin Town, Chiayi 62247, Taiwan

## Abstract

Vascular events are one of the major causes of death in case of Cushing's syndrome (CS). However, due to the relative low frequency of CS, it is hard to perform a risk assessment for these events. As represented congestive heart failure (C), hypertension (H), age (A), diabetes (D), and stroke (S), the CHADS_2_ score is now accepted to classify the risk of major adverse cardiovascular events (MACEs) in patients with atrial fibrillation. In this study, participants were enrolled from the National Health Research Institute Database (NHIRD) of Taiwan, and we reviewed 551 patients with their sequential clinically diagnosed CS data between 2002 and 2009 in relation to MACEs risk using CHADS_2_ score. Good correlation could be identified between the CS and CHADS_2_ score (AUC = 0.795). Our results show that patients with CS show significantly higher risk of vascular events and the CHADS_2_ score could be applied for MACEs evaluation. Adequate lifestyle modifications and aggressive cardiovascular risks treatment are suggested for CS patients with higher CHADS_2_ score.

## 1. Introduction

Cushing's syndrome (CS) represents the pattern of symptoms and signs caused by prolonged exposure of inappropriately high levels of the hormone cortisol [1–3]. It is relatively rare and most commonly affects adults between the ages of 20 and 50, affecting approximately 2 to 3 per million people in each year [4]. Obesity has been linked with this diagnosis. Other signs and symptoms include thinning of the reddish purple striae, plethora, proximal muscle weakness, weight gain, bruising with no obvious trauma, hypertension, diabetes, and osteoporosis [4–8]. Vascular events, either cardiovascular or cerebrovascular, are the major causes of death [4, 6, 7, 9]. However, due to the relatively low incidents of CS, it is hard to make a risk assessment for these events.

The most frequently used schema designed to stratify risk of thromboembolism in patients with atrial fibrillation is CHADS_2_ score [10–14], which closely correlate major adverse cardiovascular events (MACEs), such as myocardial infarction, stroke, and mortality risk. Clinically, CHADS_2_ score is simple to use, with one point given for congestive heart failure, hypertension, age, and diabetes and two points for stroke or transient ischemic attack. Higher score has been associated with the risk of developing MACEs and for these patients, early anticoagulation therapy should be recommended [13, 15].

Recent studies have indicated that the risks of MACEs other than atrial fibrillation have been reported and the risk assessment was performed by using CHADS_2_ [16–18]. In this study, participants with clinically diagnosed CS were adopted from the NHIRD of Taiwan. This database enrolls up to 99% of the 23 million residents of Taiwan who receive medical care through the National Health Insurance (NHI) program which consists of ambulatory and inpatient care records and the registration files of the insured. The aim of the study was to explore the relationship between CS and the risk of vascular events. Furthermore, we evaluated the predictive value of CHADS_2_ on MACEs in patients with CS.

## 2. Materials and Methods

### 2.1. Study Population

This observational study was conducted in a retrospective cohort of the Taiwanese population from the year 2002 to 2009, enrolled in NHIRD in Taiwan. The National Health Insurance Bureau of Taiwan randomly reviews the charts of 1 out of every 100 ambulatory cases and one out of every 20 inpatient cases; it also performs patient interviews to verify the accuracy of the diagnosis. In patients ≥18 years of age diagnosed with Cushing's syndrome (ICD-9-CM codes 255.0) between the years 2002 and 2009 were recruited for this study. CS patients with stroke and previous acute myocardial infarction were excluded.

### 2.2. Risk Score Calculation

The CHADS_2_ scores were calculated for each patient by assigning 1 point each for the presence of chronic heart failure, hypertension, age below 75 years, and diabetes and by assigning 2 points for history of stroke or transient ischemic attack (TIA). The study patients were divided into 4 groups by their CHADS_2_ scores: 0, 1, 2, and ≧3, respectively.

### 2.3. Study End Point and Patient Followup

Mortality data covering the years from 2002 to 2009 were used to calculate the mortality rate in each group. Each patient was tracked for 5 years from the time of their first diagnosis using administrative data to identify all patients who had MACEs such as myocardial infarction, stroke, and mortality or censored during the study period.

### 2.4. Statistical Analysis

SPSS (version 15, SPSS Inc., Chicago, IL, USA) was used for data analysis. Receiver operating characteristics curve was used to assess the prediction accuracy for MACEs by using CHADS_2_ score and plots of observed and predicted MACEs were presented. The cumulative rates of MACEs were estimated using the log rank test to examine the differences in the risk of mortality between different groups among the CS patients. The Cox proportional hazards regression model was used to compare the outcomes between different risk groups. We calculated hazard ratios (HR) along with 95% confidence intervals (CI) using a significance level of 0.05. A two-sided *P* value (*P* < 0.05) was used to determine statistical significance.

## 3. Results

There are 551 patients diagnosed with CS from 2002 to 2009 within the NHIRD. The number of patients, age, gender, and distribution of number of patients in different CHADS_2_ score groups are shown in Table 1. The mean age at diagnosis was 48 ± 20 years. 67.9% of patients were female. The mean CHADS_2_ score was 0.96 ± 1.17. Severe comorbidity (CHADS_2_ score ≧3) was noted in 10.7% in all CS patients.

**Figure 1:**
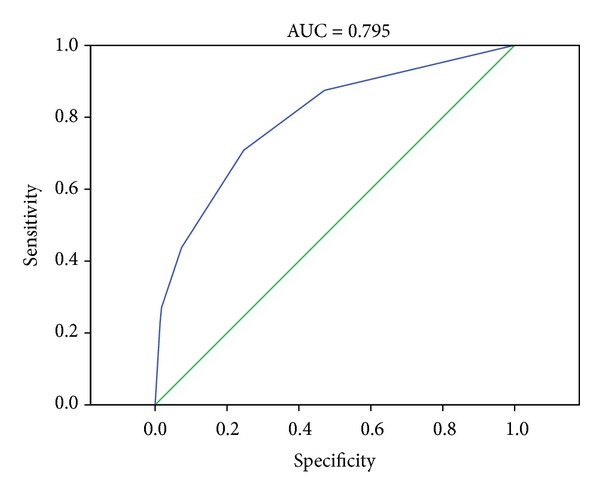
Receiver operating characteristics curve for CHADS_2_ in prediction of MACEs in Cushing's syndrome patients.

**Figure 2:**
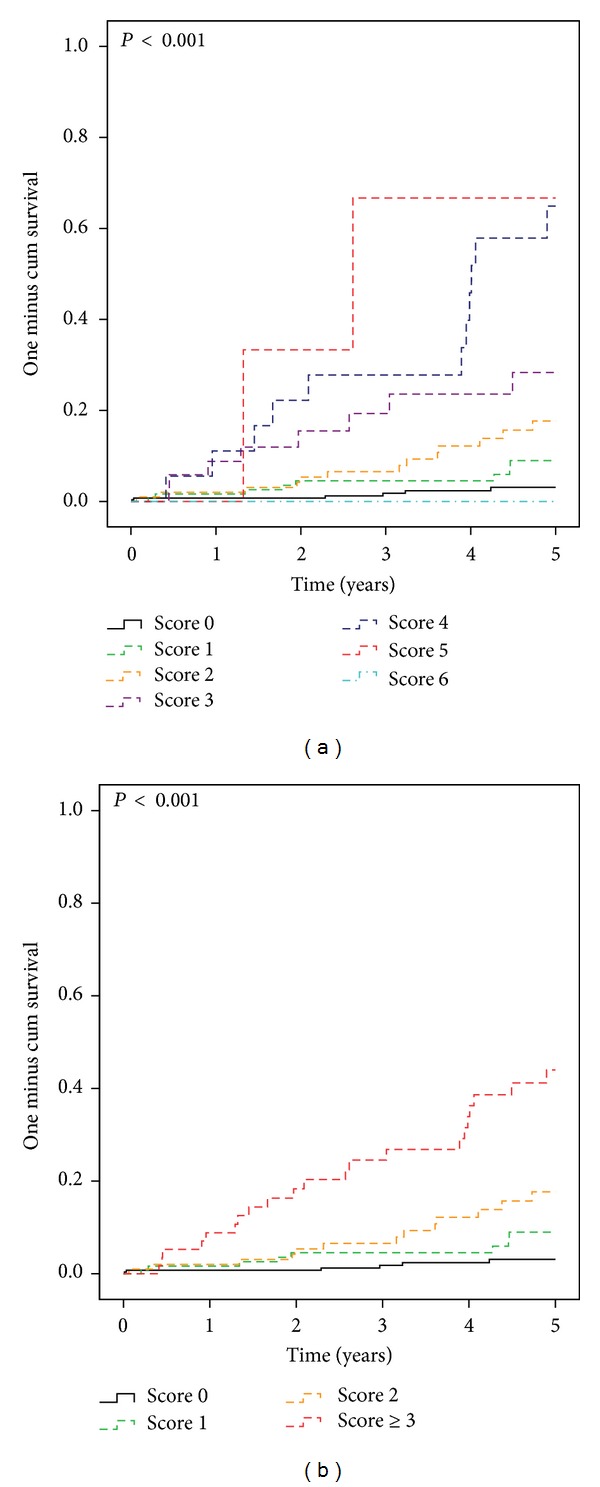
(a) MACEs risk stratified by CHADS_2_ score. (b) MACEs risk stratified by CHADS_2_ score categories.

Figure 1 showed that the* c*-statistics was 0.795 (95% CI, 0.724–0.866).  Figure 2(a) was the Kaplan-Meier survival curves. CS patients with higher CHADS_2_ score were more likely to have MACEs (*P* < 0.001). We further divided the CS patients into four groups based on the CHADS_2_ scores (Table 2). CS patients with a score of ≧3 are associated with the highest rate in MACEs (Figure 2(b), *P* < 0.001).

In multivariate analysis, each additional CHADS_2_ score is associated with 1.63-fold (95% CI, 1.29–2.06) increased risk for MACEs when CHADS_2_ score is a continuous variable (Table 3, model A). In model B of Table 3, where CHADS_2_ is an ordinal variable and divided into three groups, patients with the score ≧3 remained as an independent prognostic factor for the risk of MACEs with hazard ratios of 7.16 (95% CI, 2.31–22.15), compared with CHADS_2_ 0-1 after adjusting with other factors. As compared to male, female is associated with decreased risk for MACEs in patients with CS (model B, adjusted HR 0.45, 95% CI 0.25–0.81, and *P* = 0.009). Age is associated with increased risk for MACEs (model B, adjusted HR 1.05, 95% CI 1.00–1.05, and *P* = 0.026).

## 4. Discussion

The main finding of this study is that the CHADS_2_ score can be useful in the evaluation of the global cardiovascular risk (i.e., MACEs and death) of patients with CS. We provide the general calculation of mortality and morbidity rate as well as a risk assessment for MACEs in cases of CS. We validated the application of CHADS_2_ score in the MACEs of CS patient for the accuracy of our prediction is 79.5%.

The strengths of these data are based on the fact that it was a nationwide population-based cross-sectional study, with nearly complete follow-up information about access to healthcare institutes, as well as the fact that the dataset was routinely monitored for diagnostic accuracy by the National Health Insurance Bureau of Taiwan. The National Health Insurance (NHI) program was conducted since 1995 which requires mandatory enrollment in the government-run, universal, single-payer insurance system and provides comprehensive benefits coverage. Currently, up to 99% of the 23 million residents of Taiwan receive medical care through this program. Over 97% of the hospitals and clinics in Taiwan are contracted to provide health care services [19], which are reimbursed by the Bureau of NHI, and all data related to these services are collected and stored into the NHIRD by the National Health Research Institutes to provide a comprehensive medical care record. The data consisted of ambulatory care records, inpatient care records, and the registration files of the insured. The dataset included all claims data from Taiwan's NHI program, which was implemented as a means of financing health care for all Taiwanese citizens. The average age and gender distribution of CS patients were also compatible with a previous report [20].

There are many reports about the mortality and morbidity of CS that have been documented [21–24]. However, due to the relative rarity of CS, data collection on the incidence, mortality, and cause of death is scarce. Our results show a 8.7% overall risk of MACEs in CS patients. Poor prognosis was demonstrated in 35.6% of the patients in high risk population (CHADS_2_ ≧3), 13.0% of the CHADS_2_ = 2, 6.6% of the CHADS_2_ = 1, and 2.2% in the cases of the CHADS_2_ = 0. Current guidelines for atrial fibrillation suggest that the CHADS_2_ is useful in the selection of antithrombotic therapy; therefore, those with high risk (CHADS_2_ score ≧2) would benefit from anticoagulation therapy. For those with low (score 0) or moderate (score 1) risk, aspirin may be an alternative given that the risk of bleeding caused by anticoagulation therapy may not justify its use [14]. Antithrombotic therapy is also essential for the CS patients within the high risk population.

Our study had several limitations related to the NHIRD database. The diagnoses of CS, AMI, and stroke were dependent on ICD codes used in the NHIRD database. Another limitation is that the design of the study is retrospective. Validation of scores in retrospective cohort presents always with several limitations that weaken the strength of the results. However, we believe the relatively large number of patients may compensate for these limitations. The National Health Insurance Bureau of Taiwan, however, has made every effort to verify the accuracy of diagnosis based upon random chart reviews and patient interviews. An additional limitation of the NHIRD database was its lack of information on tobacco use, dietary habits, metabolic profiles, or other behavioral factors, which may be risk factors for AMI among CS patients. Nonetheless, given the magnitude and statistical significance of the observed effects in this study, these limitations were unlikely to have compromised our results. In fact, the strength of our study lies in its large sample size. In addition, because the NHI has 99% coverage in Taiwan, our study had minimal risk of selection bias.

## 5. Conclusion

In the present study, CHADS_2_ score could be a good tool to predict the incidence of MACEs in patients with Cushing's syndrome. Cardiovascular risks evaluation and management should be applied more aggressively for CS patients with CHADS_2_ score ≧3.

## Figures and Tables

**Table 1:** Baseline characteristics of the patients with Cushing's syndrome from 2002 to 2009 in Taiwan (*n* = 551).

Variables	Study population
	*n* (%)
Total	551 (100)
Mean age, years (±SD)	48 ± 20
CHADS_2_ score	
Mean ± SD	0.96 ± 1.17
0	271 (49.2)
1	121 (22.0)
2	100 (18.1)
≧3	59 (10.7)
Gender	
Male	177 (32.1)
Female	374 (67.9)
Hyperlipidemia	126 (22.9)
Chronic kidney disease	26 (4.7)
Coronary artery disease	42 (7.6)
Atrial fibrillation	5 (0.9)
Socioeconomic status	
Low	227 (50.3)
Moderate	195 (35.4)
High	79 (14.3)

**Table 2:** MACEs among Cushing's syndrome patients with different CHADS_2_ scores from 2002 to 2009 (*n* = 551).

Variables	Male			Female		
	*n*	Case (%)	*P* value	*n*	Case (%)	*P* value
CHADS_2_ score			<0.001			<0.001
0 (*n* = 271)	77	5 (6.5)		194	1 (0.5)	
1 (*n* = 121)	41	6 (14.6)		80	2 (2.5)	
2 (*n* = 100)	38	5 (13.2)		62	8 (12.9)	
≧3 (*n* = 59)	21	9 (42.9)		38	12 (31.6)	

**Table 3:** Hazard ratios of individual CHADS_2_ score for MACEs in patients with Cushing's syndrome (*n* = 551).

	Model A∗			Model B∗∗		
	Adjusted HR	95% CI	*P* value	Adjusted HR	95% CI	*P* value
CHADS_2_ score	1.63	1.29–2.06	<0.001			
0				1		
1				1.95	0.63–6.03	0.246
2				2.77	0.90–8.48	0.074
≧3				7.16	2.31–22.15	0.001
Gender						
Male	1			1		
Female	0.50	0.28–0.90	0.022	0.45	0.25–0.81	0.009
Age	1.03	1.00–1.05	0.006	1.02	1.00–1.05	0.026
Hyperlipidemia	0.50	0.23–1.08	0.078	0.55	0.26–1.15	0.114
Chronic kidney disease	1.26	0.52–3.03	0.599	1.35	0.58–3.14	0.484
Coronary artery disease	1.44	0.67–3.09	0.343	1.59	0.76–3.32	0.216
Atrial fibrillation	1.17	0.24–5.53	0.843	1.17	0.25–5.47	0.839
Socioeconomic status						
Low	1			1		
Moderate	1.69	0.90–3.17	0.099	1.57	0.84–2.93	0.156
High	1.22	0.32–4.54	0.764	1.17	0.31–4.32	0.812

Adjusted HR: adjusted hazard ratio; 95% CI: 95% confidence interval.

∗Model A: CHADS_2_ score as continuous variable.

∗∗Model B: CHADS_2_ score as ordinal variable.
